# Knowledge, attitudes, and practice regarding medication use in pregnant women in Southern Italy

**DOI:** 10.1371/journal.pone.0198618

**Published:** 2018-06-19

**Authors:** Monica Navaro, Luigi Vezzosi, Gabriella Santagati, Italo Francesco Angelillo, Collaborative Working Group

**Affiliations:** Department of Experimental Medicine of the University of Campania “Luigi Vanvitelli”, Naples, Italy; National Institute of Health, ITALY

## Abstract

The study assessed knowledge, attitudes, and medication use of a random sample of pregnant women attending outpatient Gynecology and Obstetrics clinics at randomly selected public General and Teaching hospitals in Naples, Italy. A total of 503 women participated. Those more likely to know that a pregnant woman with chronic condition must discuss whether or not to take a medication with the physician were Italian, aged 31–40 years, employed, with no history of abortion, having had a medical problem within the previous year, with a better self-perceived health status, who knew how to use medications during pregnancy, and who needed information on medications. The knowledge of the potential risk of using non-prescribed medications during pregnancy was significantly higher in employed women, who received information from physicians, who knew how to use medications during pregnancy, and who knew the possible damages related to medications use. More than half had used at least one medication. Those aged 26–35 years, Italian, non-graduated, in the third trimester, having had a medical problem within the previous year, with a risky pregnancy, and with a knowledge that women with chronic condition must discuss whether or not to take a medication with the physician were more likely to use medication. Less than half had used medication without a physician’s advice. Those who were more likely to self-medicate were older, Italian, multiparous, with no history of abortion, who knew that women with chronic condition must discuss whether or not to take a medication with the physician, who did not know the potential risk of using non-prescribed medication during pregnancy, who had used prescribed medication during pregnancy, and who needed information about medications. Educational programs for women about medication use are important to increase their knowledge of the potential risks to the pregnant women and the unborn child in order to reduce self-medication.

## Introduction

It has become evident that the use of medications, either with or without physician’s prescription and over-the-counter, among pregnant women have increased in the past years all over the world [1,2]. Medications use may be due because the population frequently become pregnant with conditions which require continuous or episodic therapy [3,4] or pregnancy-induced medical conditions with the need of pharmacological treatment [5,6]. There is evidence that inappropriate medications use during pregnancy may put the mother at greater potential risk for several maternal and unborn child adverse outcomes [7,8]. In this context, proper management of medications use is of utmost public health importance and should be high on the agenda for health policymakers.

In recent years few epidemiological investigations on medications use during pregnancy have been conducted [2,9–12]. In Italy previous studies showed that the prevalence of pregnant women using at least one medication was 48% after excluding vitamin and mineral products [13], 63.1% excluding supplements of iron and vitamins [14], and 70.4% excluding vitamins, mineral supplements, iron, and herbal or complementary medication products [3]. However, studies specifically addressing the level of knowledge, the attitudes, and the medications use among pregnant women, to the best of authors’ knowledge, have been not located in Italy to date. Acquiring this information no doubt could be useful for counseling and developing strategies/policies in order to support pregnant women to make informed decisions since, in Italy, the prenatal healthcare is provided free of charge in public and private accredited hospitals by delivering evidence-based interventions at four critical times during pregnancy. Therefore, the present investigation was undertaken to provide an examination regarding utilization of medications from a large sample of pregnant women in Italy. Specifically, the objectives were to characterize knowledge, attitudes, and medications use during the pregnancy and additionally to investigate which characteristics are associated with these outcomes.

## Patients and methods

### Setting and sample

The cross-sectional study was undertaken from May 2016 through March 2017 in Naples, Italy. Three public General Hospitals and one Teaching Hospital were selected randomly from the list of the 16 hospitals that provides childbirth services in the geographic area. The number of births per year in the selected facilities were respectively 2200, 830, 720, and 690. All pregnant women who presented for their routine clinic appointment at the outpatient Gynecology and Obstetrics clinics of the selected hospitals were eligible.

The sample size was determined by using a single population proportion formula considering the following assumptions: the prevalence of women who use medications during pregnancy without the prescription of a physician should be about 45%, a confidence interval of 95%, and a margin of error of 5%. The final sample size was calculated to be 475, accounting for an assumed non-response rate of 20%.

### Data collection

The Ethics Committee of the Teaching Hospital University of Campania “Luigi Vanvitelli” reviewed and approved the study protocol and questionnaire.

The Directors of the hospitals received a letter informing about the survey and explaining the purpose and the methodology. Permission to carry out the study was obtained before commencement.

Pregnant women attending the outpatient clinics were approached by three trained research assistants, unaffiliated with the health staff, and asked whether they would participate in the survey. The research assistants collected the data through a face-to-face pre-tested questionnaire in the waiting room. Data collection was conducted after explanation to each woman background, objectives, data protection, and privacy. All participants, before answering, signed an informed consent form explaining the study procedures and that they were free to leave out questions if they did not wish to answer. The participants were assured that all information was kept confidential, because no names were recorded. The participants did not receive any compensation for participating.

### Instrument

The standardized questionnaire began with a preamble of the study and the length of the interview was approximately 20 minutes. The questionnaire consisted of 30 questions in 5 sections and is found in S1 File. The first section explored the socio-demographic (i.e., age, marital status, education level, employment status) and medical data (i.e., self-assessed general state of health, medical history, gravidity, parity, gestational age, number of pregnancies, number of visits to the physician). A high risk pregnancy was defined as a pregnancy in which are present existing health conditions, such as diabetes, high blood pressure, kidney disease, or being HIV-positive, overweight, obesity, multiple birth, substance abuse, young or old maternal age, or toxic exposures. The second section was used to measure the level of knowledge about specific aspects of medications use during pregnancy, including the possibility of harm to the unborn (e.g., fetal growth retardation, intrauterine death, malformations) and to their health, and the potential risk regarding the use of non-prescribed medications. The third section was used to assess whether respondents would be willing or not to use medications while pregnant without the prescription of a physician. Participants were also asked the reason(s) of their attitude. The fourth section was used to evaluate the practice, by asking if the women had used medications, not including vitamin, mineral supplements, and herbal treatment (i.e., health problem for the use, compliance to dose regimens, duration of treatment), with or without the prescription during the current pregnancy. Drugs were classified according to the World Health Organization Anatomical Therapeutic Chemical (ATC) classification system. The fifth section was used to evaluate the source(s) of information on medications use during pregnancy and the interest in learning more.

The questionnaire was pretested among 20 subjects for face and construct validity and if questions were interpreted as intended. This pilot study confirmed women’s understanding, feasibility of completion, and resulted in few modifications of the wording of the questions to ensure that they were easily comprehensible.

### Statistical analysis

Data (S2 File) analysis was performed using the Stata statistical software [15]. First, frequencies, proportions, and summary statistics were used to describe the sample in relationship to the relevant variables. Second, bivariate analysis was carried out to assess whether each of the exploratory characteristics was associated with the dependent variable. Variables with a *p*-value ≤ 0.25 were further analyzed with logistic regression using *p* at 0.2 for entry and *p* at 0.4 for removal as criteria for backward and forward variable selection. Third, multivariate stepwise logistic regression was performed to identify the independent contribution of each variable with the following outcomes of interest: knowledge that a woman with chronic condition must discuss whether or not to take a medication with the physician during pregnancy (Model 1); knowledge about the potential risk of using non-prescribed medications during pregnancy (Model 2); use of at least one medication in the current pregnancy (Model 3); and practiced at least one self-medication in the current pregnancy (Model 4). In the models the outcome variables were dichotomized as follows: the knowledge was measured by grouping the responders who answered correctly that a pregnant woman with chronic health condition must discuss whether or not to take a medication with the physician versus the others (Model 1) and by grouping those who correctly answered that there is a potential risk of using non-prescribed medications and the need to receive a medical advice during pregnancy versus the others (Model 2); the behaviors in the current pregnancy were measured by grouping responders who had used at least one medication versus the others (Model 3) and those who had practiced at least one self-medication versus the others (Model 4). All multivariate regression models included socio-demographic (age, nationality, marital status, educational level, employment status), medical (history of abortion, parity, gestational age, high risk pregnancy, self-perceived health status, at least one health problem in the last year), and knowledge (physicians as a source of information, need of additional information about medications use in pregnancy, knowledge of the possible harms to the unborn and of the possible damages to the women due to the use of medications during pregnancy, knowledge regarding how to use medications according to the trimester of the pregnancy) variables. The following variables were also included: knowledge that a woman with chronic health condition should know whether or not to use a medication during pregnancy in Models 2–4; knowledge about the potential risk of using non-prescribed medications in Models 1, 3, and 4; and having used at least one medication with prescription during pregnancy in Model 4. Odds ratios (ORs) and 95% confidence intervals (CIs) were used when measuring the association of the different characteristics with the outcomes of interest.

All tests were two sided and degree of statistical significance was declared at a *p* value ≤ 0.05.

## Results

### Characteristics of the sample

Of the 510 pregnant women approached, 503 participated for a response rate of 98.6%. Ages varied between 15 and 44 years, 40.8% had a secondary educational level, almost 60% had at least one child, almost half were in the third trimester, and 17.9% had a pregnancy at risk (Table 1).

**Table 1:** Main socio-demographic and personal characteristics of the study population.

	n	%
**Age (*years*)**	29.1±6.4 (15–44)*	
≤20	55	10.9
21–25	100	19.9
26–30	126	25.1
31–35	124	24.6
>35	98	19.5
**Nationality**		
Italian	403	80.1
Other	100	19.9
**Marital status**		
Married/Cohabiting	427	84.9
Other	76	15.1
**Education level**		
No formal education or elementary school	52	10.3
Middle school	188	37.4
High school	205	40.8
College degree or higher	58	11.5
**Employment status**		
Employed	224	44.6
Unemployed	279	55.4
**Parity**		
Nulliparous	202	40.2
Parous	301	59.8
**Number of pregnancies not completed**^a^		
0	317	63
1	144	28.6
≥2	42	8.4
**Gestational age (*weeks*)**	26.1±9*	
First trimester	42	8.4
Second trimester	225	44.7
Third trimester	236	46.9
**Pregnancy at risk**		
Yes	90	17.9
No	413	82.1
**Self-perceived health status**	7.5±1.7 (2–10)*	
**At least one health problem in the last year**		
Yes	90	17.9
No	413	82.1
**At least one medical consultation in the last year**		
Yes	462	91.9
No	41	8.1
**At least one General Practitioner visit in the current pregnancy**		
Yes	133	26.4
No	370	73.6
**Use of medications in previous pregnancies**^b^		
Yes	217	57.9
No	158	42.1
**Use of medications in the current pregnancy**		
Yes	300	59.6
No	203	40.4
**Use of at least one medication with prescription**		
Yes	205	40.8
No	298	59.2
**Use of at least one medication without prescription**		
Yes	221	43.9
No	282	56.1
**Number of medications used**^**c**^	1.9±1 (1–7)*	
1	125	41.7
2	114	38
≥3	61	20.3

*Mean ± standard deviation (range)

^a^Miscarriage or abortion

^b^Only for those who had other pregnancies completed or not completed (n = 375)

^c^Only for those who have taken medications in the current pregnancy (n = 300)

### Knowledge towards medication use

The majority (74.7%) were aware that a woman with chronic health condition must discuss whether or not to use a medication with the physician during pregnancy, 81.1% knew the possibility of harm to the unborn due to medications use during pregnancy (e.g., fetal growth retardation, intrauterine death, malformations), 41.9% knew the possibility of damage to their health, and 83.1% recognized the potential risk of using non-prescribed medications during pregnancy and the need to receive a medical advice.

### Behavior regarding medication use

The prevalence of medications use during the current pregnancy was 59.6% and the use with and without physician’s advice at least once was 40.4% and 43.9%, respectively. Medications were commonly used in the first (23.8%) and second (40.3%) trimesters, with a number ranging from 1 to 7. Among the users, respectively 41.7%, 38%, and 20.3% took one, two, and three or more medications. Among those who had used a medication, 67.7% and 73.7% had used with and without physician’s advice, respectively. The main health problems that required a medication with the prescription of a physician were obstetric disorders (28%), urinary tract infection symptoms and other infections (17.4%), digestive disorders (8.3%), hypo/hyperthyroidism (8.7%), fever/common cold symptoms (8.7%), and hypertension (7%). The main reasons for using medication without physician’s advice were a not serious disease (47%), advice/information by pharmacists (29.7%), they knew that the medication is safe during pregnancy (14.6%), and emergency care (13.5%). The most frequent reasons for non-prescribed medication use were fever/common cold symptoms (32.6%), headache/migraine (29.5%), digestive disorders (18.2%), and nerve pain (15.5%).

Considering all trimesters, according to the ATC classification system, the two most commonly classes of medications prescribed were anti-infective agents for systemic use and for genitourinary system and reproductive hormones (Table 2). The most commonly prescribed medications were progesterone (14.6%), amoxicillin (8.7%), levothyroxine (8.3%), fosfomycin (7.3%), acetylsalicylic acid (7.3%), methyldopa (5.7%), and paracetamol (5.3%). The most commonly classes of medications used for self-medication were for nervous system and for alimentary tract and metabolism. The most frequently medications self-used were paracetamol (69.7%) and aluminum hydroxide (10%).

**Table 2:** Use of prescribed and non-prescribed medications by trimester of pregnancy and by pharmacological class according to ATC code level 1 of the study population.

**Trimester of pregnancy**	**Overall** **(Total n = 558)**		**Prescribed** **(Total n = 300)**		**Non-prescribed** **(Total n = 258)**	
	**N**	**%**	**n**	**%**	**n**	**%**
**1**^**st**^	133	23.8	88	29.3	45	17.4
**2**^**nd**^	225	40.3	104	34.7	121	46.9
**3rd**	79	14.2	36	12	43	16.7
**All**	54	9.7	52	17.3	2	0.8
**Unknown**	67	12	20	6.7	47	18.2
ATC class^*^	**Overall** **(Total n = 558)**		**Prescribed** **(Total n = 300)**		**Non-prescribed** **(Total n = 258)**	
	**N**	**%**	**n**	**%**	**n**	**%**
**A**	89	16	41	13.7	48	18.6
**B**	40	7.2	40	13.3	-	-
**C**	21	4	22	7.3	1	0.4
**G**	44	7.9	44	14.7	-	-
**H**	43	7.7	43	14.4	-	-
**J**	71	12.8	66	22	5	1.9
**M**	20	3.5	4	1.3	16	6.2
**N**	207	37.1	27	8.9	180	69.8
**R**	20	3.6	12	4.1	8	3.1
**V**	1	0.2	1	0.3	-	-

^*^ **ATC class A:** Alimentary tract and metabolism–**B:** Blood and blood forming organs–**C:** Cardiovascular system–**G:** Genitourinary system and reproductive hormones–**H:** Systemic hormonal preparations (excluding reproductive hormones and insulin)–**J:** Anti-infective for systemic use–**M:** Musculoskeletal system–**N:** Nervous system–**R:** Respiratory system–**V:** Various ATC structures

### Attitudes towards medication use

Among participants who had not used medications during the current pregnancy, 85.1% indicated that they would not consider use a medication without physician’s prescription and the main reasons were that they would wait their advice (77.8%), concerns on the risk for the unborn baby (52.8%), and concerns about adverse reactions (39%). Whereas, the women were willing to self-medicate in case of a non-serious health problem (86.7%), a recommendation by the pharmacist (38.7%), and an emergency (26.7%).

### Multivariate logistic regression analysis

Multivariate logistic regression analyses were conducted to estimate the strength of the independent associations between several factors and the different outcomes of interest while controlling for all other variables in the Models. Table 3 presents the statistically significant factors associated with the different outcomes of interest. The first multivariate logistic regression model examined the association between several characteristics of the respondents and their knowledge that a woman with chronic health condition must discuss whether or not to use a medication with the physician during pregnancy. The results revealed a statistically significant association between this knowledge and several maternal factors. Indeed, this knowledge was higher among Italian women, subjects of 31 to 40 years, employed, with no history of abortion, who have had a medical problem in the previous year, and with a better self-perceived health status. Moreover, pregnant women who know how to use medications according to the trimester of the pregnancy, and who need additional information about using medications in pregnancy have higher chances of gaining adequate knowledge (Model 1).

**Table 3:** Results of the multivariate logistic regression analysis identifying the variables significantly associated with the different outcomes of interest.

Variable	OR *(*95% CI)	*p* value
**Model 1.** Knowledge that a pregnant woman with a chronic health condition must discuss whether or not to take a medication with the physician		
Log likelihood = -208.13, χ^2^ = 152.18 (18 df), *p*<0.0001		
Need of additional information about using medications in pregnancy	2.59 (1.55–4.34)	<0.001
Self-perceived health status	1.33 (1.34–1.55)	<0.001
At least one health problem in the last year	4.88 (1.75–13.59)	0.002
Age (*years*)		
≤20	1*	
31–35	2.92 (1.18–7.23)	0.02
36–40	3.5 (1.27–9.69)	0.016
History of abortion	0.57 (0.34–0.95)	0.031
Employment status	1.79 (1.05–3.05)	0.032
Nationality	1.88 (1.01–3.5)	0.044
Correct knowledge how to use medications according to the trimester of pregnancy	1.95 (1.01–3.76)	0.045
**Model 2.** Knowledge of the potential risk of using non-prescribed medications and the need to receive a medical advice during pregnancy		
Log likelihood = -193.6, χ^2^ = 69.8 (8 df), *p*<0.0001		
Employment status	2.54 (1.42–4.55)	0.002
Correct knowledge of the possible damages to the women due to the use of medications in pregnancy	2.44 (1.35–4.44)	0.003
Physicians as a source of information about using medications in pregnancy	2.72 (1.37–5.39)	0.004
Correct knowledge how to use medications according to the trimester of pregnancy	2.49 (1.2–5.19)	0.015
Physicians as a source of information on the risk for the unborn baby due to the use of medications in pregnancy	1.82 (1.04–3.17)	0.036
**Model 3.** Had used at least one medication		
Log likelihood = -257.1, χ^2^ = 164.28 (17 df), *p*<0.0001		
Gestational age (*trimester*)		
Second	1*	
Third	2.36 (1.51–3.69)	<0.001
High-risk pregnancy	6.03 (2.77–13.1)	<0.001
Correct knowledge that a pregnant woman with a chronic health condition must discuss whether or not to take a medication with the physician	2.53 (1.49–4.3)	0.001
Age (*years*)		
≤20	1*	
26–30	2.27 (1.26–4.1)	0.006
31–35	2.75 (1.44–5.26)	0.002
At least one health problem in the last year	2.81 (1.41–5.58)	0.003
Educational level		
College degree or higher	1*	
No formal education or elementary school	3.53 (1.25–9.97)	0.017
Middle school	2.53 (1.09–5.85)	0.03
High school	2.99 (1.39–6.43)	0.005
Nationality	2.25 (1.24–4.09)	0.007
**Model 4.** Had practiced at least one self-medication		
Log likelihood = -287.43, χ2 = 115.02 (13 df), *p*<0.0001		
Correct knowledge about the potential risk of using non-prescribed medications in pregnancy	0.34 (0.19–0.61)	<0.001
At least one medication with prescription in the current pregnancy	2.45 (1.62–3.7)	<0.001
Parity	2.01 (1.29–3.14)	0.002
Nationality	2.19 (1.25–3.83)	0.006
Correct knowledge that a pregnant woman with a chronic health condition must discuss whether or not to take a medication with the physician	2.05 (1.19–3.52)	0.009
Age (*years*)		
≤20	1*	
>40	14.68 (1.42–151.68)	0.024
History of abortion	0.63 (0.41–0.96)	0.032
Employment status	1.55 (1.02–2.37)	0.04
Need of additional information about using medications in pregnancy	1.52 (1.01–2.31)	0.049

* Reference category

The correct knowledge about the potential risk of using non-prescribed medications and the need to receive a medical advice during pregnancy was significantly higher in women employed, in those who knew the use of medications according to the trimester of pregnancy, who knew the possible damages to the woman herself due to the use of medications during pregnancy, and in those who stated that physician was the main source of information about medications use in pregnancy and about the risk for the unborn baby due to the use of medications during pregnancy (Model 2 in Table 3).

The use of at least one medication in the current pregnancy was statistically significantly associated with several maternal factors. Italian women, aged 26 to 35 years, with a low level of education, with a pregnancy at risk, in the third trimester, and those who have had a medical problem in the previous year were significantly more likely to had used at least one medication in their current pregnancy. Moreover, correct knowledge that a pregnant woman with chronic health condition must discuss whether or not to take a medication with the physician increases the probability of using at least one medication in the current pregnancy (Model 3 in Table 3).

Italian women, those older than 40 year-old, employed, pluriparous, with no history of abortion, who had used at least one medication with prescription in the current pregnancy, who know that a pregnant woman with chronic health condition must discuss whether or not to take a medication with the physician, those with a lower knowledge about the potential risk of using non-prescribed medications during pregnancy, and who need additional information about using medications in pregnancy were significantly more likely to use medication without prescription during pregnancy (Model 4 in Table 3).

### Sources of information

Of all women, 81.3% had received information about medications use during pregnancy and physicians were the most common source (75.3%), followed by internet (46.9%), and pharmacists (14.7%), whereas, only half had received information on the risk for the fetus (52.3%) and the majority of them from Gynecologists (86.3%). Finally, 57.8% would welcome in learning more about medications use during pregnancy.

## Discussion

This survey used a large sample of pregnant women to examine their knowledge, attitudes, and behaviors toward medications use and their associated factors and provides important information that can guide intervention and activities by policy-makers and health professionals.

Among this sample, 59.6% used medications during the current pregnancy. This finding is comparable to the 64% in Ethiopia [16], 64% [2] and 59% [17] in Canada, but lower than the 85.2% in Scotland [18], 93.9–88.8% [1] and 82.5% [9] in the United States, 81.2% in Europe, North and South America and Australia [3], 76.4% in the United Kingdom [12] and 69.1% in Norway [19]. Moreover, the prevalence was higher than the 49% in Iceland [20], 46.8% in Ireland [10], 27% in Canada [2], 26.5% in Australia [4], and 17.9% in Western, Northern, and Eastern Europe, North America, and Australia [6]. In this study, 43.9% women reported nonprescription medication use. This value was lower than those reported in Europe, America and Australia with 66.9% [3] and in Australia with 57.5% [4]; whereas it was higher than the 23% in Texas [21], 19.5% in Ireland [22], and 12.5% in Holland [23]. It is interesting to observe that the prevalence of self-medication is considerably lower than the 69.2% for medications [24] and higher than the 32.7% for antibiotics [25] observed in the general population in the same area. The lower value in this sample compared with the general population may partly be explained by the fact that pregnant women pay more attention to the medication use. This is supported by the observation that non-seriousness of the illness was the response identified by the sample as major motivation for using medications without physician advice and, furthermore, that almost one-third had received advice/information by pharmacists. The role of the pharmacists may be important, since they are often the first health care provider that pregnant women visit to request advice/information, in order to address misconceptions regarding the use of medications. This finding is in concordance with present literature [11,24,26].

It is important to note that, according to multivariate logistic regression analysis, some of the findings pertaining to the socio-demographic variables were in the expected direction. Indeed, a key conclusion to be drawn is that the occupation, an important socio-economic indicator, significantly influenced the respondent’s knowledge since when compared with women who did not have an occupation, those from higher socio-economic strata have more adequate knowledge about the potential risks of using non-prescribed medications during pregnancy. Result of the current study is also generally consistent with a previous study identifying occupation associated with the level of knowledge [27]. Knowledge of the sample that a woman with chronic health condition should know whether or not to take a medication during pregnancy significantly varied based on their nationality. Italian women were more likely to be knowledgeable when compared to the non-Italian. This might be a consequence of an easier access to health services. This could also be partly explained by the finding that Italian women were more than 2 times as likely to use medications as non-Italian. This result is in line with previous research [3]. The results regarding the medications use either with or without physician’s advice showed that this behavior was significantly less frequent among the women with college degree and above. The current findings do corroborate other research [12,16]. Moreover, those younger were less likely to use medications and these findings are consistent with similar studies [2,10,22]. Younger and older women have different medications behavior and this may be based on the assumption that those older have likely already gained experience on medications use.

Specific knowledge of the women who participated in the survey on the different aspects of medications use during pregnancy, including the possibility of harm to the unborn and to their health, and the potential risk regarding the use of non-prescribed medications, revealed a lack of sufficient knowledge. Indeed, only 41.9% knew the possibility of damage to their health and this is alarming. Therefore, identifying trusted sources of information is extremely important and it is not unexpected that three-quarters indicated their health care providers as trustworthy resource. Other studies are in agreement with this finding [12,28–32]. It is important to underline that preconception counseling between women and health care workers are needed in order to increase the level of knowledge. This is supported by the fact that the knowledge about potential risk of using non-prescribed medications during pregnancy was significantly higher in those who had received information by physicians. Therefore, physicians should be engaged in delivering key messages, since they have an important role during women’s pregnancy in providing support with up-to-date evidence-based information about safety of medications used during pregnancy and in ensuring adequate and safe maternity care. On the other hand, more than half of the sample expressed a desire to learn more on medications use during pregnancy and those who have this need were more likely to use medication without prescription. Moreover, one of the more interesting finding was that the women were more likely to self-medicate without knowing the potential risk of using non-prescribed medications during pregnancy. Acknowledging the great need for information indicates that these women would benefit from educational intervention and, therefore, it is essential to expand the role of the healthcare providers. Ideally, a woman who is planning pregnancy should consult her health care provider well before she becomes pregnant. Care during pregnancy is a team effort involving an obstetrician and a primary care provider and they play a pivotal role in solving the problem of poor information regarding the use of medications in pregnancy, since a pregnant woman should check with her doctor before taking any drug.

The important issue of the information and education was also stressed by the result that the progesterone was the most commonly used medications and the value, 14.6%, was higher than those reported in other countries mainly in the United States with values ranging from 0.4% [10] to 4.5% [1], whereas it was consistent with the 11.7% observed in the already mentioned study conducted in Italy [14]. This finding seem to highlight a still disturbing practice and suggest that, although progesterone therapy did not result in a significant higher rate of live births among women with a history of abortion [33], important information on the risks and potential benefits of medications in this population is missing. Therefore, again the necessity for pregnant women to routinely receive from healthcare workers evidence-based information in order to avoid the risk of unnecessary use of medications.

When interpreting these results, it is important to highlight that there are certain limitations that might impact upon the conclusions drawn. First, the observational nature of the study limits the ability to identify the chronology of events, although several associations have been observed. Second, because all information was assessed by self-report and could not be checked, patients’ responses may have been subject to reporting bias. Surveys are inherently limited by response bias, in which respondents may answer questions how they *should* practice instead of how they *actually* practice. The estimates are likely to be an underestimation of the true prevalence of medications use. However, there is little reason to think the data would be subject to social desirability or recall biases also because an anonymous survey has been used. Third, this research was conducted among participants recruited in a city located in Southern Italy and it is possible that their knowledge, attitudes, and medications use differ from those of pregnant women in other parts of the country and, therefore, the generalizability of the findings to population in other geographic areas may need to be established. We believe, however, that the findings found in this sample are similar regardless of geographic location. Fourth, half of the selected women were in the third trimester and this means that the remaining women may not have had the chance yet to use medication, for instance to treat conditions usually occurring in late pregnancy. However, our interest was to characterize the medications use during all period of pregnancy and the sensitivity analysis restricted to women having full overview of the pregnancy period has been performed and no substantially differences have been observed. Notwithstanding these limitations, there are several strengths to this study, the greatest of which is that it is the first Italian data regarding knowledge, attitudes, and medication use among pregnant women. Moreover, the high response rate and the inclusion of a representative sample of the population provide important insights into knowledge, attitudes, and practices regarding medications use.

To conclude, these findings clearly indicate the need for increased communication of women around the use of medications during pregnancy in order to increase their knowledge and to reduce self-medication.

## Supporting information

S1 File This is the questionnaire in English. (DOCX)

S2 File This is the dataset. (XLSX)
