# Supplementary material for: Knowledge, attitudes, and practice regarding medication use in pregnant women in Southern Italy
Source: PLoS One. 2018 Jun 19;13(6):e0198618. doi: 10.1371/journal.pone.0198618 (PMC6007931; doi:10.1371/journal.pone.0198618)
Supplement: S1 File — (DOCX) [file pone.0198618.s001.docx]

**QUESTIONNAIRE**

**A. SOCIO-DEMOGRAPHIC AND HEALTH-RELATED CHARACTERISTICS**

*I will ask you some questions to gather information about your socio-demographic and health-related characteristics*

**A1. How old were you on your last birthday?** _____

**A2. What is your nationality?** □ Italian □ Other *(specify)* ______________________

**A3. What is your marital status?** □ Married □ Single (never married) □ Separated\Divorced\Widowed □ Other *(specify)* ___________

**A4. What is the highest educational level you have completed?** _____________________________

**A5. What is your occupation?**________________________________________

**A6.** (*if the respondent does not have a husband or a partner, go to question A7)* **What is the current occupation of your husband/partner?** __________________

**A7. How many sons do you have?** ______

**A8. How many deliveries have you had?** __________

**A9. Have you experienced a miscarriage or an abortion?** □No □Yes, how many times? ____________

**A10. In which pregnancy week are you?** _______________

**A11. Do you have any medical problem?** □ No □ Yes *(specify)* _______________________________________

**A12. How would you rate your health status on a 1 to 10 scale, with 1 meaning bad at all and 10 very good health status?**

Bad 1 2 3 4 5 6 7 8 9 10 Very good

**B. ACCESS TO THE HEALTH CARE SERVICES**

*I will ask you some questions to gather information about your access to the health care services*

**B1. In the previous twelve months, have you had a health problem?** □ No □ Yes (specify)_________________

**B2.** **In the previous twelve months, have you had visited a physician?** □ No *(go to section C)*

□ Yes, who? ________________________________ how many times?__________________________

**B3**. **Why did you have visited a physician?** (more than one answer is allowed)

**□** Check-up, how many times during this pregnancy? ______________

**□** Check of chronic diseases (*specify)* **_________________** how many times in the previous twelve months? _________

**□** Medical prescription, how many times in the previous twelve months? ______________

**□** Other *(specify)* _______________________________ how many times in the previous twelve months? _______

**C. MEDICATIONS USE DURING PREGNANCY**

*I will ask you some questions to gather information about your medication use during this pregnancy and in previous pregnancies*

**C1.** *(if the respondent is primiparous go to C2)* **Did you take any medications in previous pregnancies?** □ No □ Yes

**C2.** **Did you take any medications during this pregnancy?** □ No □ Yes

*(if the respondent answered No to question, go to Section D)*

**C3. What medication did you use during your current pregnancy?**

*For each medication I will ask you some information regarding the reason for using, how long you have used it, if you used it even before pregnancy and if your physician prescribed the medication*

| **PRESCRIBED MEDICATION USE** | | | | | | | | | | |
| --- | --- | --- | --- | --- | --- | --- | --- | --- | --- | --- |
| **Which medication did you use?** | | **For which health condition?** | | **Which was the dose regimen and how long did you use the medication?** | | **Did you start this therapy before or during pregnancy?**  *(specify the week)* | | **How long did you use the medication before pregnancy?** | **Did you stop using the medication during pregnancy?** | **Did you change the prescribed dose on your own?** |
| **1** | |  | |  | |  | |  | **□ no □ yes; why?** | **□ no □ yes; why?** |
| **2** | |  | |  | |  | |  | **□ no □ yes; why?** | **□ no □ yes; why?** |
| **3** | |  | |  | |  | |  | **□ no □ yes; why?** | **□ no □ yes; why?** |
| **4** | |  | |  | |  | |  | **□ no □ yes; why?** | **□ no □ yes; why?** |
| **NON-PRESCRIBED MEDICATION USE** | | | | | | | | | | |
| **Which medication did you use?** | **For which health condition?** | | **Which was the dose regimen and how long did you use the medication?** | | **Did you start this therapy before or during pregnancy?**  *(specify the week)* | | **How long did you use the medication before pregnancy?** | | **Did you stop using the medication during pregnancy?** | |
| **1** |  | |  | |  | |  | | **□ no □ yes; why?** | |
| **2** |  | |  | |  | |  | | **□ no □ yes; why?** | |
| **3** |  | |  | |  | |  | | **□ no □ yes; why?** | |
| **4** |  | |  | |  | |  | | **□ no □ yes; why?** | |

*(if the respondent used a medication during pregnancy only with a prescription of a physician, go to Section D).*

**C4. Why did you use medications without the prescription of a physician?** (specify the reason for each medication)

A) The illness was too mild B) Availability of an old prescription C) Advice/information by pharmacists

D) Emergency E) The physician was not available F) The physician did not prescribe it

medication 1 _____________, reason _________________________________________________________

medication 2 _____________, reason _________________________________________________________

medication 3 _____________, reason _________________________________________________________

medication 4 _____________, reason_________________________________________________________

**D. KNOWLEDGE TOWARDS MEDICATION USE** **DURING PREGNANCY**

*I will ask you some questions to explore your knowledge related to medications use during pregnancy*

**D1. The medications used for a chronic disease should be modified during pregnancy?**

□ No

□ A woman should know whether or not to use a medication

□ The use must be suspended/stopped because it is harmful for the unborn baby

□ The use should be suspended/stopped because it is harmful for the woman

□ Other (specify) ____________________________

**D2. A medication can be used at any stage of pregnancy?**

□ No, never

□ Only in the first trimester □ Only in the second trimester □ Only in the third trimester □ At any time of pregnancy

**D3.** **Which are the possible effects of medications use during pregnancy for the unborn baby?**

|  | Yes | No | Do not know |
| --- | --- | --- | --- |
| **2.1** Fetal growth retardation |  |  |  |
| **2.2** Intrauterine death |  |  |  |
| **2.3** Chromosomal abnormalities |  |  |  |
| **2.4** Visible malformations in subsequent ages |  |  |  |
| **2.5** Other (specify) ______________________________________ |  |  |  |

**D4. Which are the possible effects of medications use during pregnancy for the woman?**

|  | Yes | No | Do not know |
| --- | --- | --- | --- |
| **3.1** Abortion |  |  |  |
| **3.2** Uterine contraction |  |  |  |
| **3.3** Bleeding |  |  |  |
| **3.4** Postpartum depression |  |  |  |
| **3.5** Loss of memory |  |  |  |
| **3.6** Other (specify) ______________________________________ |  |  |  |

**D5. Non-prescribed medications can be used during pregnancy?** (more than one answer is allowed)

□ Yes, they have less side effects □ Yes, the pharmacist must provide all necessary advices and information

□ No, they are potentially harmful □ No, the physician’s advice is necessary

□ Other (specify) ________________________

**E.ATTITUDES TOWARDS MEDICATIONS USE DURING PREGNANCY**

*I will ask you some questions to gather information about your attitudes toward medications use during pregnancy*

**E1.** *(ask this question only to respondent who did not use non-prescribed medication during this pregnancy or in the previous pregnancies)*

**Would you take medications during pregnancy without the prescription of a physician?**

| **□ no** *(more than one answer is allowed)* | **□ yes** (more than one answer is allowed) |
| --- | --- |
| □ I have fear of the side effects | □ The health problem is not serious |
| □ I will wait a medical consultation | □ I have an old medical prescription |
| □ I use only natural products | □ An emergency sasassaaraauuuurgenzaurga_____________________________________________________________________________________________________________________________ |
| □ I have concern about the risk for the unborn baby | □ I am traveling |
| □ I have concern about the risk for my health | □ The physician is not available |
| □ I have already used it and I have had problems | □ Pharmacist’s advice |
| □ Other _______________________________ | □ Friends and relatives’ advice |
|  | □ The physician does not prescribe it |
|  | □ The physician is not easily accessible (times and distance) |
|  | □ Knowledge about the side effects |
|  | □ Previous experience of using medications in pregnancy without a physician’s prescription |
|  | □ Other ____________________________________________ |

**F. SOURCES OF INFORMATION**

*I am going to ask you some questions to know the sources and need of information about medications use in pregnancy*

**F1.** **From which sources did you receive information about medications in pregnancy?** (more than one answer if possible)

**□** None **□** Physicians □ Midwives □ Pharmacists □ Internet □ TV □ Newspapers □ Other (specify) ___________________

**F2. Have you ever received information from a physician about the potential health risks for your unborn baby due to the medications use in pregnancy?**

□ No □ Yes, from who? _____________________________________________________________

**F3. Do you usually read the information leaflet of the medications you use?**

□ No □ Yes

**F4.** **How would you rate the utility of the "Pregnancy and lactation" section of the information leaflet on a 1 to 10 scale, with 1 meaning not useful and 10 very useful?**

Not useful 1 2 3 4 5 6 7 8 9 10 Very Useful

**F5. Do you feel you need more information about the use of medications in pregnancy?**

□ No □ Yes
